# Thermal Thresholds and Genetic Sensitivity in Barrel Racing Performance of Quarter Horses Across Temperature–Humidity Indices

**DOI:** 10.1111/jbg.70056

**Published:** 2026-06-05

**Authors:** Mário Luiz Santana, Annaiza Braga Bignardi

**Affiliations:** ^1^ Grupo de Melhoramento Animal de Mato Grosso (GMAT), Instituto de Ciências Agrárias e Tecnológicas Universidade Federal de Rondonópolis (UFR) Rondonópolis MT Brazil

**Keywords:** genetic parameters, genotype by environment interaction, heat stress, random regression, reaction norm

## Abstract

Heat stress is an increasingly important challenge for the performance and welfare of equine athletes, particularly in competitions conducted across diverse climatic conditions. Understanding the genetic basis of performance responses to increasing thermal load is therefore essential to support robust genetic evaluation and sustainable selection strategies. The objective of this study was to evaluate barrel racing performance of Brazilian Quarter Horses across thermal environments defined by two widely used indicators, the temperature–humidity index (THI) and the wet bulb globe temperature (WBGT), and to estimate genetic parameters associated with baseline performance and environmental sensitivity. A total of 351,993 barrel racing time (BRT) records from 13,960 Quarter Horses were analyzed. Segmented regression models were applied to least squares means to identify heat stress thresholds, while random regression models with polynomial functions were used to estimate covariance components, genetic parameters, and correlations along the thermal gradients. Distinct thermal thresholds were identified for both indices, at THI = 74.7 and WBGT = 23.6, beyond which performance deteriorated more rapidly. Heat stress conditions were observed in 31.15% and 20.71% of barrel racing events according to the THI and WBGT thresholds, respectively, emphasizing the practical relevance of the evaluated thermal gradients. Random regression models assuming continuous thermal variation provided the best overall fit to the data. Additive genetic variance remained relatively stable across thermal environments, whereas permanent environmental and residual variances increased under higher thermal load. Mean heritability under thermoneutral conditions was approximately 0.21 for THI and WBGT, declining modestly to 0.17–0.18 under extreme heat stress. Genetic correlations between thermoneutral and extreme environments remained high for both indices (at approximately 0.97), indicating strong genetic continuity of performance and no substantial reranking of estimated breeding values across the thermal gradient. Overall, THI and WBGT yielded highly consistent results in terms of thresholds, genetic parameters, and selection outcomes. Although WBGT showed slightly greater sensitivity under extreme conditions, THI offered smoother response patterns and clear operational advantages due to its simplicity and ease of calculation. No evidence of substantial genotype‐by‐environment interaction was detected for BRT. However, the results suggest that incorporating genetic tolerance to heat stress as a complementary selection criterion may help sustain barrel racing performance under increasingly challenging climatic conditions.

## 1 Introduction

Equestrian sports represent an important sector of the global animal production and sports industry, combining athletic performance, cultural relevance and substantial economic value (Adelman and Thompson 2017). Among the different equine disciplines, barrel racing is one of the most popular speed events, particularly in the Americas. The competition consists of completing a cloverleaf pattern around three barrels in the shortest possible time, requiring exceptional acceleration, agility, balance and coordination (Freitag et al. 2025; Santana et al. 2025).

Barrel racing is characterized by short‐duration but extremely high‐intensity exercise. Horses are exposed to substantial anaerobic demands, rapid increases in heart rate, elevated muscular activity and marked thermogenic responses (Binda et al. 2016; Gomes et al. 2020; Souza et al. 2018). Under favourable climatic conditions, these physiological responses are efficiently regulated. However, competitions may be held outdoors, and environmental conditions vary widely across events. In warm and humid environments, which are common in tropical and subtropical regions, the dissipation of metabolic heat becomes less efficient. As a result, horses may experience elevated thermal load, requiring additional physiological adjustments such as increased sweating, peripheral vasodilation and altered cardiovascular function. These compensatory responses can increase energetic costs and potentially impair athletic performance, even during short races (Brownlow and Mizzi 2022; Ebisuda et al. 2023; McConaghy et al. 2002).

Despite the clear biological plausibility that thermal conditions influence equine performance, scientific evidence addressing this issue in sport horses remains limited. Most studies in horses have focused on acute physiological responses to exercise under heat stress, including changes in body temperature, blood metabolites and sweating rate (Brownlow and Mizzi 2023; Ebisuda et al. 2023; Klous et al. 2020). While informative, these studies generally overlook inter‐individual differences and do not address whether such differences have a genetic basis. Consequently, little is known about genetic tolerance to heat stress in horses, particularly in the context of competitive performance.

Reaction norm models provide a suitable quantitative‐genetic framework to address these issues. These models describe phenotypic performance as a function of a continuous environmental variable, allowing simultaneous estimation of genetic merit under baseline conditions and genetic sensitivity to environmental change (Carabaño et al. 2019). In studies of heat stress, temperature–humidity indices are commonly used to represent environmental load, as they integrate ambient temperature and humidity into a single biologically meaningful measure (Bohmanova et al. 2007). Reaction norm models enable the assessment of genetic variation in both overall performance and thermal tolerance, as well as changes in genetic variance and heritability across the environmental gradient (Dolores Gómez et al. 2015).

Although reaction norm approaches have been widely applied in livestock species to study heat stress tolerance, their application to equine sports is scarce (Bartolomé et al. 2018; Dolores Gómez et al. 2015). To date, no study has evaluated genetic tolerance to heat stress in barrel racing horses using this framework. Therefore, the objective of this study was to evaluate the performance of Quarter Horses in barrel racing across thermal environments defined by two widely used thermal‐load indicators: the temperature–humidity index (THI), which is extensively applied in livestock species, and the wet bulb globe temperature (WBGT), which has been widely adopted in equine science. Segmented regression and reaction norm models were applied to barrel racing time records to identify thermal thresholds and to estimate genetic parameters associated with baseline performance and environmental sensitivity.

## 2 Materials and Methods

### 2.1 Data and Quality Control

Racing performance records from 13,960 Quarter Horses were obtained from the Sistema de Gerenciamento de Provas (SGP) and comprised 2855 barrel racing events conducted between 2010 and 2024 across 23 Brazilian states, representing all major geographic regions of the country. The largest proportion of records originated from the states of São Paulo (61.1%), Paraná (13.7%), Rio de Janeiro (7.6%), Minas Gerais (5.6%) and Mato Grosso (2.6%), reflecting the regional concentration of organized barrel racing competitions in Brazil.

The phenotypic trait analysed was barrel racing time (BRT), expressed in seconds and defined as the total time required for a horse–rider pair to complete the official three‐barrel cloverleaf pattern. According to standard competition rules, a penalty of 5 s is added to the recorded time for each barrel displaced during the run. However, explicit information regarding penalties was not available in the database. To minimize the inclusion of potentially penalized performances, a conservative data‐editing strategy was adopted based on prior exploratory analyses and previously established criteria. Specifically, only BRT records up to 4.99 s above the Brazilian national record time of 16.109 s were retained, thereby excluding performances that were likely affected by penalties. This criterion was applied following the approach described by Santana et al. (2025). In addition, records were removed from races with fewer than 10 competing horses and from horses with missing sire or dam information. Further quality control procedures included the exclusion of records from horses younger than 36 months or older than 144 months at the time of competition, restricting the analysis to animals within a biologically and competitively relevant age range. Rider effects were also controlled by removing records associated with riders who participated in fewer than three races, thereby reducing the influence of poorly represented rider–horse combinations on model estimation.

After quality control, the final analytical dataset included 351,993 records. A comprehensive summary of the edited data structure, including the number of animals, records, and pedigree depth, is presented in Table 1. Additional details regarding data characteristics and the racing circuit are provided in Santana et al. (2025).

**TABLE 1:** Summary of data structure for barrel racing time of Quarter Horses.

Statistics	Values
Horses in the pedigree file, *n*	31,639
Records, *n*	351,993
Horses with records, *n*	13,960
Mean number of records per horse, *n*	25.21
Horses with up to 10 records, *n*	5930
Horses with 11–20 records, *n*	2889
Horses with 21–30 records, *n*	1589
Horses with 31 to 40 records, *n*	975
Horses with 41–50 records, *n*	662
Horses with more than 50 records, *n*	1915
Male records, *n*	172,680
Female records, *n*	179,313
Stallions with progeny record, *n*	2099
Mares with progeny record, *n*	7188
Races (contemporary groups), *n*	12,790
Riders, *n*	5253
Mean and standard deviation of the barrel racing time, s	17.977; 0.735
Minimum and maximum of the barrel racing time, s	16.109; 21.098
Mean and standard deviation of the temperature, °C	22.946; 3.752
Minimum and maximum temperature, °C	7; 35
Mean and standard deviation of the humidity, %	69.779; 13.509
Minimum and maximum humidity, %	20; 95
Mean and standard deviation of the THI, unit	70.588; 5.345
Minimum and maximum of the THI, unit	47.042; 83.026
Mean and standard deviation of the WBGT, unit	19.796; 3.461
Minimum and maximum of the WBGT, unit	5.299; 28.295
Municipalities, *n*	295

Abbreviations: THI = temperature–humidity index, WBGT = the wet‐bulb globe temperature.

### 2.2 Weather Data

Given the broad geographic distribution of the competitions, meteorological information was derived from the National Aeronautics and Space Administration Prediction of Worldwide Energy Resources (NASA POWER) satellite‐based system (https://power.larc.nasa.gov/). The barrel racing events analysed in this study were conducted in 295 distinct localities across Brazil, encompassing a wide range of climatic conditions. In this context, the use of ground‐based meteorological stations is limited by the insufficient spatial coverage of the Brazilian weather station network and by the presence of temporal gaps in historical records, particularly over the extended period during which the races were held. Previous studies have demonstrated that NASA POWER offers accurate, continuous, and spatially consistent climatic information for agricultural and livestock applications in Brazil, including assessments of heat stress (Carrara et al. 2023; Monteiro et al. 2018). A key advantage of NASA POWER over conventional meteorological networks is the availability of uninterrupted daily time series, which ensures homogeneity in environmental covariates across all records.

For each barrel racing record, daily meteorological variables were retrieved from NASA POWER based on the date of the event and the geographic coordinates of the corresponding racetrack. This procedure allowed location‐specific environmental conditions to be assigned to individual performance records, ensuring consistency in the characterization of thermal environments across the diverse set of competition sites analysed in this study.

### 2.3 Thermal Indices

Two established thermal indices were employed to characterize the environmental thermal load to which horses were exposed during competition and to evaluate genetic variation in tolerance to heat stress. These indices were selected based on their widespread use in animal science and their biological relevance to thermoregulation, particularly in equine athletes. The first index considered was the temperature–humidity index (THI), calculated according to the formulation proposed by the United States National Research Council (NRC). Daily mean air temperature (T, °C) and relative humidity (RH, %) measured at 2 m above ground were combined as follows:

$$\mathrm{THI}=\left(1.8\times T+32\right)-\left(0.55-\left(0.0055\times \mathrm{RH}\right)\times \left(1.8\times T-26\right)\right).$$

The second index evaluated was the wet bulb globe temperature (WBGT), which is widely adopted in equine science and is recommended by the International Equestrian Federation as an indicator of heat stress risk during athletic activities. The WBGT was calculated following the definition originally proposed by Schroter and Marlin (1995):

$$\text{WBGT}=\left(0.7\times T_{\mathrm{wb}}\right)+\left(0.3\times \mathrm{BGT}\right),$$

where $T_{\mathrm{wb}}$ represents the wet‐bulb temperature of the ambient air and BGT denotes the black globe temperature. Wet‐bulb temperature was calculated from air temperature and relative humidity following Stull (2011). BGT was not directly available from NASA POWER and was therefore estimated using an empirical regression based on *T*, total incoming shortwave solar radiation, and wind speed, following the framework described by Liljegren et al. (2008).

In addition to the thermal conditions on the day of competition, potential delayed effects of heat stress were also considered. Previous studies in dairy cattle and horses have demonstrated that thermal load experienced on the day of production or competition and during preceding days can exert cumulative effects on animal performance (Carabaño et al. 2014; Hammami et al. 2013; Santana et al. 2026). Based on this evidence, each thermal index was calculated as the daily average on the race day and for the two preceding days, using moving averages relative to the date of each event. This approach allowed the evaluation of both immediate and short‐term carryover effects of thermal load on barrel racing performance. Because the database did not include reliable information on the exact time of each run, these daily thermal indices were used to provide a standardized characterization of the thermal environment across all records. Although this approach does not capture short‐term thermal peaks at the exact time of competition, it is consistent with the objective of evaluating day‐level and short‐term cumulative effects of thermal load, as horses are commonly housed at the competition site before and during events. Finally, Pearson correlation coefficients were computed between THI and WBGT to quantify the degree of association between the two indices and to assess their similarity in characterizing thermal conditions across events.

### 2.4 Performance in Barrel Racing Across Thermal Indices

Least squares means of BRT were obtained to describe phenotypic performance patterns across classes of thermal indices. These estimates were derived using a linear model fitted in R, including the random effects of horse and residual, and the fixed effects of contemporary group, sex, animal age at competition (modelled as linear and quadratic covariates), and thermal index (THI or WBGT). Contemporary groups were defined by the combination of event, category, subcategory and competition date, reflecting the organizational structure of barrel racing events and ensuring appropriate control of systematic environmental and management effects.

A segmented linear regression model was applied to the least squares means of BRT across THI and WBGT values to identify potential heat stress thresholds. The ‘segmented’ R package (Muggeo 2003) was used to fit linear models with slope discontinuities at statistically identified breakpoints (BP). The segmented linear regression model can be described as follows:

$$\begin{array}{c} y_{q}=a+b_{1}X_{q}+e_{q},\text{when}\;X_{q}\leq \mathrm{BP}\\ y_{q}=a+b_{1}X_{q}+b_{2}\left(X_{q}-\mathrm{BP}\right)+e_{q},\text{when}\;X_{q}>\mathrm{BP} \end{array}$$

where $y_{q}$ was the least squares mean of the BRT corresponding to the *q* ^th^ THI or WBGT value; $X_{q}$ was the *q* ^th^ THI or WBGT value; a was the intercept; $b_{1}$ and $b_{2}$ were the regression coefficients (slopes) of the response variable on THI or WBGT before and after the BP (heat stress threshold), respectively; and $e_{q}$ was the residual term.

### 2.5 Genetic Analysis

The covariance components and genetic parameters were estimated using eight animal models: two repeatability models and six random regression models. All models included the same set of systematic and random effects and differed only in the way the additive genetic, permanent environmental, and residual components were modeled with respect to the thermal index, considering either THI or WBGT.

The two repeatability models differed in the specification of the residual variance, which was assumed to be heterogeneous according to the heat stress threshold identified in the previous step using THI (M0) or WBGT (M1). For thermal index values less than or equal to the threshold, the residual variance corresponding to thermoneutral conditions was assumed, whereas for values above the threshold, the residual variance associated with heat stress conditions was considered.

Two random regression (reaction norm) models were subsequently fitted using a threshold‐based thermal load function, as previously defined, considering either THI (M2) or WBGT (M3):

$$\begin{array}{c} y_{\text{iklm}}=\beta_{0}+\beta_{1}A_{\text{iklm}}+\beta_{2}A_{\text{iklm}}^{2}+\beta_{3}f\left(I_{\text{iklm}}\right)+a_{i0}+a_{i1}f\left(I_{\text{iklm}}\right)+pe_{i0}\\ \;+pe_{i1}f\left(I_{\text{iklm}}\right)+b_{k}+cg_{l}+s_{m}+e_{\text{iklm},} \end{array}$$

where $y_{\text{iklm}}$ was the BRT record of the i‐th horse; $A_{\text{iklm}}$ was the age of the horse at the competition date (months), fitted as linear ($\beta_{1}$) and quadratic ($\beta_{2}$) covariates; $\beta_{0}$ was the overall intercept; $\beta_{3}$ was the fixed regression coefficient on thermal load; $f\left(I_{\text{iklm}}\right)$ was the thermal load function defined as $f\left(I_{\text{iklm}}\right)=\max\left(0,I_{\text{iklm}}-I_{0}\right)$, where $I_{\text{iklm}}$ was the thermal index (THI or WBGT) matched to the record and $I_{0}$ was the threshold value identified in the previous step; $a_{i0}$ and $a_{i1}$ were the additive genetic random regression coefficients for the intercept (thermoneutral performance) and slope (genetic sensitivity to thermal load), respectively; $pe_{i0}$ and $pe_{i1}$ were the permanent environmental random regression coefficients for the intercept and slope, respectively, accounting for repeated records and persistent non‐genetic differences among animals across competitions; $b_{k}$ was the random effect of rider; $cg_{l}$ was the fixed effect of contemporary group, accounting for event‐specific systematic effects at the time of competition, including differences among events, categories, and competition dates; $s_{m}$ was the effect of sex; and $e_{\text{iklm}}$ was the residual term. A heterogeneous residual variance structure with two classes was adopted based on the previously defined heat stress threshold according to THI or WBGT values.

The remaining four models were random regression models of additive genetic and permanent environmental effects on the thermal index. Models M4 and M5 used linear Legendre polynomials fitted on THI and WBGT, respectively, whereas models M6 and M7 used quadratic Legendre polynomials fitted on THI and WBGT, respectively. The models can be described as follows:

$$\begin{array}{c} y_{\text{iklm}}=\beta_{0}+\beta_{1}A_{\text{iklm}}+\beta_{2}A_{\text{iklm}}^{2}+\sum_{q=0}^{n}\beta_{q}^{\left(I\right)}\phi_{q}\left(I_{\text{iklm}}^{*}\right)\\ \;+\sum_{q=0}^{n}a_{\mathrm{iq}}\phi_{q}\left(I_{\text{iklm}}^{*}\right)+\sum_{q=0}^{n}pe_{\mathrm{iq}}\phi_{q}\left(I_{\text{iklm}}^{*}\right)\\ \;+b_{k}+cg_{l}+s_{m}+e_{\text{iklm}} \end{array}$$

where $y_{\text{iklm}}$ was the BRT record of the i‐th horse; $\beta_{0}$ was the overall intercept; $\beta_{1}$ and $\beta_{2}$ were the fixed regression coefficients describing the population‐level linear and quadratic effects of age at competition ($A_{\text{iklm}}$, in months), respectively; $I_{\text{iklm}}$ denoted the thermal index matched to the record (THI or WBGT), and $I_{\text{iklm}}^{*}$ was the corresponding standardized value of $I_{\text{iklm}}$ scaled to the interval $\left[-1,1\right]$; $\phi_{q}\left(I_{\text{iklm}}^{*}\right)$ was the q‐th Legendre orthogonal polynomial evaluated at $I_{\text{iklm}}^{*}$; $\beta_{q}^{\left(I\right)}$ were the fixed regression coefficients describing the population mean trajectory of performance across the thermal gradient; $a_{\mathrm{iq}}$ were the random regression coefficients for the additive genetic effect of the i‐th horse across the thermal gradient; $pe_{\mathrm{iq}}$ were the random regression coefficients for the permanent environmental effect of the i‐th horse across the thermal gradient; $b_{k}$ was the random effect of rider; $cg_{l}$ was the systematic effect of contemporary group; $s_{m}$ was the systematic effect of sex; and $e_{\text{iklm}}$ was the residual term. The polynomial order was set to 𝑛 = 1 for the linear model and to 𝑛 = 2 for the quadratic model. The residual variance was assumed to be heterogeneous with two classes, as described above for the other models.

All analyses were conducted under a Bayesian framework. Markov chain Monte Carlo sampling consisted of 450,000 iterations, with a burn‐in period of 100,000 iterations and a thinning interval of 25, resulting in 14,000 posterior samples used for inference. Convergence and mixing of the chains were visually assessed through inspection of trace plots. Default priors implemented in the BLUPF90 family of programs were adopted for all analyses, assuming flat priors for systematic effects and inverse‐Wishart priors for covariance components. Estimation of covariance components and genetic parameters was performed using the GIBBSF90+ program (Misztal et al. 2014).

### 2.6 Model Comparison

Model fit was evaluated and compared using the deviance information criterion (DIC) (Spiegelhalter et al. 2002). The comparison aimed to assess the relative adequacy of models based on THI, a thermal index widely adopted across livestock species, and WBGT, which is more commonly applied in equine studies, for describing performance variation and genetic responses to thermal conditions. Lower DIC values indicate a better balance between model fit and complexity, thereby supporting the identification of the most appropriate thermal index and modelling strategy for genetic evaluation under variable thermal environments. It is important to highlight that threshold‐based and polynomial random regression models differed not only in the scaling of the thermal covariate but also in their functional assumptions. The former modelled changes only above the estimated heat stress threshold, whereas the latter allowed continuous responses across the entire thermal gradient. Therefore, model comparison was interpreted in terms of both functional form and model parameterization.

### 2.7 Implications for Selection

After identifying the best‐fitting model based on THI and the best‐fitting model based on WBGT, the posterior means of estimated breeding values (EBV) were evaluated for stallions with at least 10 progeny records for barrel racing time. For each stallion 𝑖, EBV along the thermal gradient was obtained as follows:

$${\mathrm{EBV}}_{i}=t^{\prime }{\hat{u}}_{i},$$

where *t* was the vector of orthogonal polynomial coefficients evaluated at a given value of the thermal index (THI or WBGT), and ${\hat{u}}_{i}$ was the vector of posterior mean EBV corresponding to the random regression coefficients of horse *i*. For polynomial random regression models, EBV were interpreted at specific values of the thermal gradient rather than directly from individual random regression coefficients, because the intercept represents the center of the standardized environmental scale. Breeding values were predicted under thermoneutral conditions, defined as thermal index values below the identified threshold, and under heat stress conditions, defined by the maximum THI or WBGT value observed in the dataset. Based on these EBV, the top 5% and 10% of animals were selected separately under each thermal condition. Selection consistency was evaluated under two complementary perspectives. First, within the same thermal index (THI or WBGT), the proportion of stallions selected in common was quantified between thermoneutral and heat stress conditions. Second, across thermal indices, the proportion of stallions selected in common was assessed by comparing THI‐ and WBGT‐based selections under the same thermal condition.

To evaluate whether animals identified as genetically more tolerant to heat stress also differed in overall genetic merit for BRT under a conventional evaluation framework, an auxiliary analysis was conducted following a procedure conceptually similar to that of Bohmanova et al. (2005). EBV from the repeatability model that did not account for changes in performance across the thermal gradient (M0) was used as the reference evaluation. Independently, results from random regression models with linear Legendre polynomials fitted on THI (M4) and WBGT (M5) were used specifically to rank animals with at least 20 barrel racing time (BRT) records according to their EBV for response to thermal load. For each of these models, the 100 most thermotolerant and the 100 least thermotolerant animals were identified, and the mean difference in EBV from the reference model (M0) was calculated between these two groups.

## 3 Results

### 3.1 Distribution of Performance Records Across Thermal Indices

**FIGURE 1:**
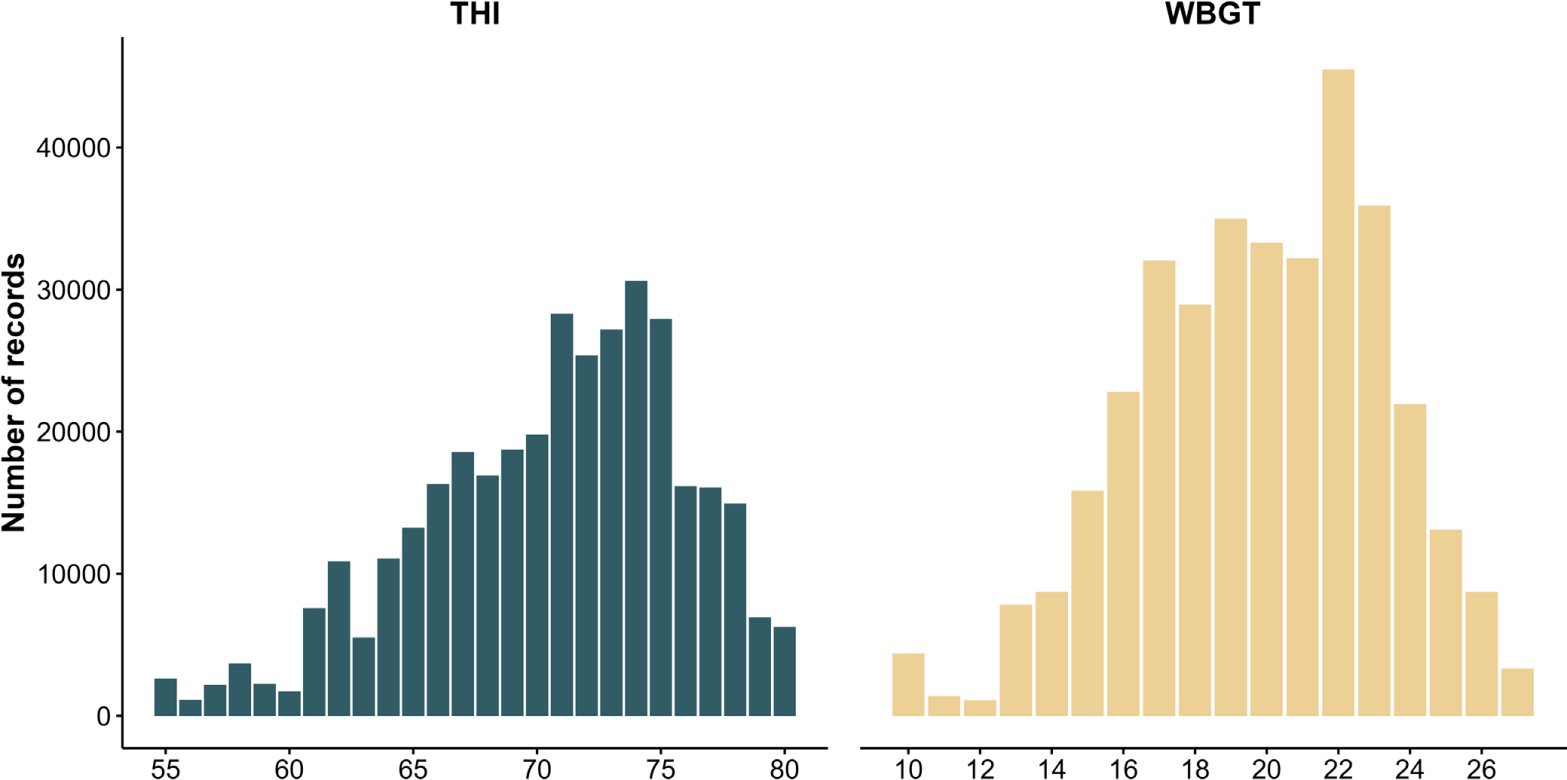
Distribution of barrel racing time records according to the values of the temperature–humidity index (THI) and the wet‐bulb globe temperature (WBGT). [Colour figure can be viewed at wileyonlinelibrary.com]

**FIGURE 2:**
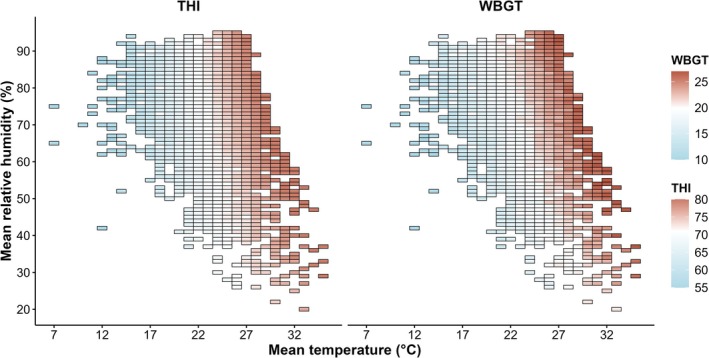
Mean temperature, relative humidity and corresponding temperature–humidity index (THI) and the wet‐bulb globe temperature (WBGT) around race days. [Colour figure can be viewed at wileyonlinelibrary.com]

Figure 1 illustrates the distribution of performance records according to the values of the THI and WBGT. Records were predominantly concentrated within intermediate ranges of both indices, with progressively fewer observations toward the lower and upper extremes of the thermal gradients. Across all observations, the Pearson correlation coefficient between THI and WBGT was 0.960 (*p* < 0.001), with a 95% confidence interval ranging from 0.959 to 0.960. Consistent with this association, Figure 2 depicts the mean ambient temperature and relative humidity averaged over the day of the race and the two preceding days, together with the corresponding mean values of THI and WBGT observed in the dataset. The distributions show that similar combinations of temperature and humidity translated into closely aligned THI and WBGT values across the range of environmental conditions represented.

### 3.2 Heat Stress Threshold and Barrel Racing Performance Across Thermal Indices

**FIGURE 3:**
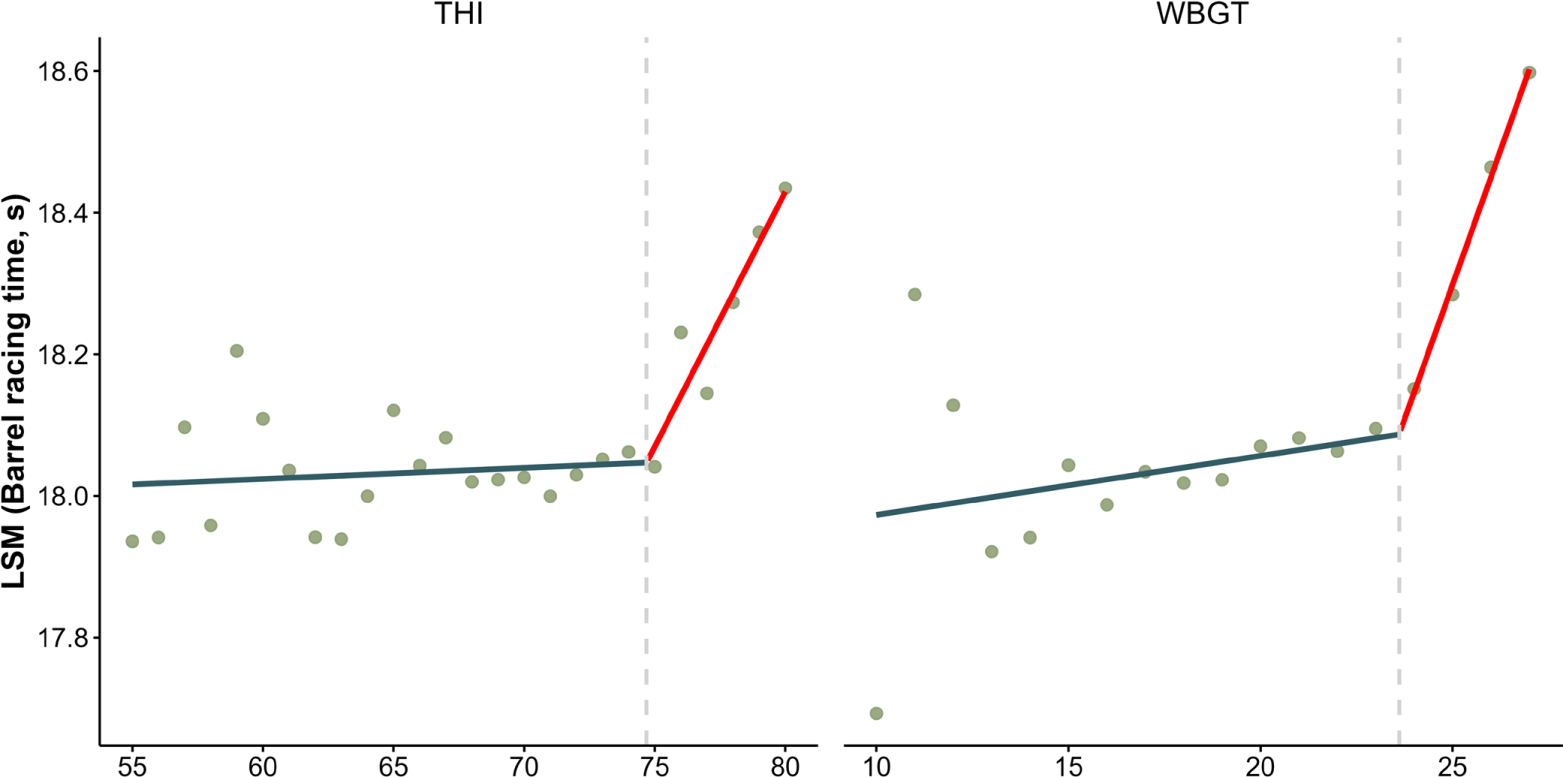
Least squares means (LSM, circles) of barrel racing performance and fitted segmented regression models (solid line) across temperature–humidity index (THI) and the wet‐bulb globe temperature (WBGT) values. [Colour figure can be viewed at wileyonlinelibrary.com]

Across both thermal indices, the segmented models identified distinct breakpoints along the environmental gradients, with comparatively smaller slope estimates below the thresholds and larger slope estimates beyond the thresholds, as reflected in the fitted least squares means trajectories (Figure 3). For the THI, the segmented model explained a substantial proportion of the variation in BRT performance, with an adjusted R‐squared of 0.712. The estimated heat stress threshold was 74.687 (*p* < 0.001), with a standard error (SE) of 0.895. The estimated intercept was 17.930 (SE = 0.174). Below the threshold, the slope estimate was 0.002 (SE = 0.003). Above the threshold, the slope increased to 0.070 (SE = 0.017), reflecting a steeper change in performance with increasing THI beyond the identified breakpoint. For the WBGT, the segmented regression yielded an R‐squared of 0.651. The estimated heat stress threshold occurred at 23.609 (SE = 0.943, *p* < 0.05). The intercept estimate was 17.890 (SE = 0.136). Prior to the threshold, the slope was 0.008 (SE = 0.008), whereas after the threshold, the slope increased to 0.144 (SE = 0.055).

### 3.3 Geographic Distribution of Records Across Brazil and Events Conducted Under Heat Stress Conditions

**FIGURE 4:**
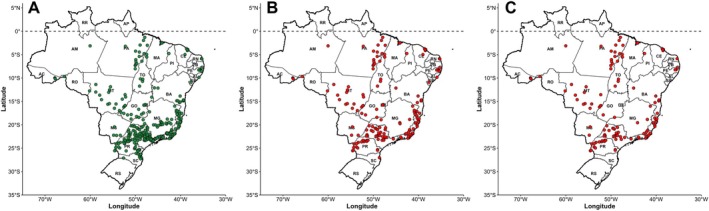
Geographic distribution of barrel racing events across Brazil (A) and occurrence of competitions under thermal stress conditions based on temperature–humidity index (THI, B) and wet‐bulb globe temperature (WBGT, C) thresholds. [Colour figure can be viewed at wileyonlinelibrary.com]

Only three Brazilian states did not host barrel racing competitions: Rio Grande do Sul (RS), located in the extreme south of the country, and Roraima (RR) and Amapá (AP), both located in the extreme north. Performance records were obtained from 295 distinct municipalities, encompassing a total of 2825 competitive events and demonstrating broad geographic and environmental coverage across Brazil (Figure 4A). Of the total number of events conducted nationwide, 880 events (31.15%) occurred under mean THI values above the identified threshold, distributed across 161 municipalities (54.58%) (Figure 4B). In addition, 585 events (20.71%) were held in 119 municipalities (40.34%) with mean WBGT values exceeding the corresponding threshold (Figure 4C). From the total number of barrel racing time (BRT) records (*n* = 351,993), 84,682 records (24.06%) were collected under THI conditions above the heat stress threshold. For WBGT, 44,082 records (12.52%) were obtained on days classified as heat stress conditions according to the identified WBGT threshold.

### 3.4 Model Comparison

Overall, the ranking of models was consistent across thermal indices, with continuous polynomial specifications outperforming segmented and repeatability approaches in terms of model fit (Table 2). Among all specifications, models assuming continuous environmental variation with quadratic polynomial functions yielded the lowest DIC values. The model fitted with THI and a quadratic polynomial (M6) showed the best overall fit (DIC = 691), followed by the corresponding WBGT quadratic model (M7; DIC = 843). Models based on linear polynomial functions also performed better than segmented and repeatability models, with lower DIC values for both thermal indices (M4 and M5). In contrast, models incorporating a segmented thermal load function exhibited substantially higher DIC values for both THI (M2) and WBGT (M3). The repeatability models (M0 and M1) showed the highest DIC values, indicating the poorest fit among the evaluated alternatives.

**TABLE 2:** Comparison of alternative models evaluated for barrel racing performance across thermal indices.

Model	Thermal index	Functional form	Environmental structure	DIC
M6	THI	Quadratic polynomial	Continuous	691
M7	WBGT	Quadratic polynomial	Continuous	843
M5	WBGT	Linear polynomial	Continuous	1392
M4	THI	Linear polynomial	Continuous	1502
M2	THI	Thermal load function	Segmented	2215
M3	WBGT	Thermal load function	Segmented	2858
M1	WBGT	Repeatability	—	3610
M0	THI	Repeatability	—	3717

Abbreviations: DIC = deviance information criterion, expressed as original values minus 1,948,000, THI = temperature–humidity index, WBGT = the wet‐bulb globe temperature.

### 3.5 Genetic Parameters

**FIGURE 5:**
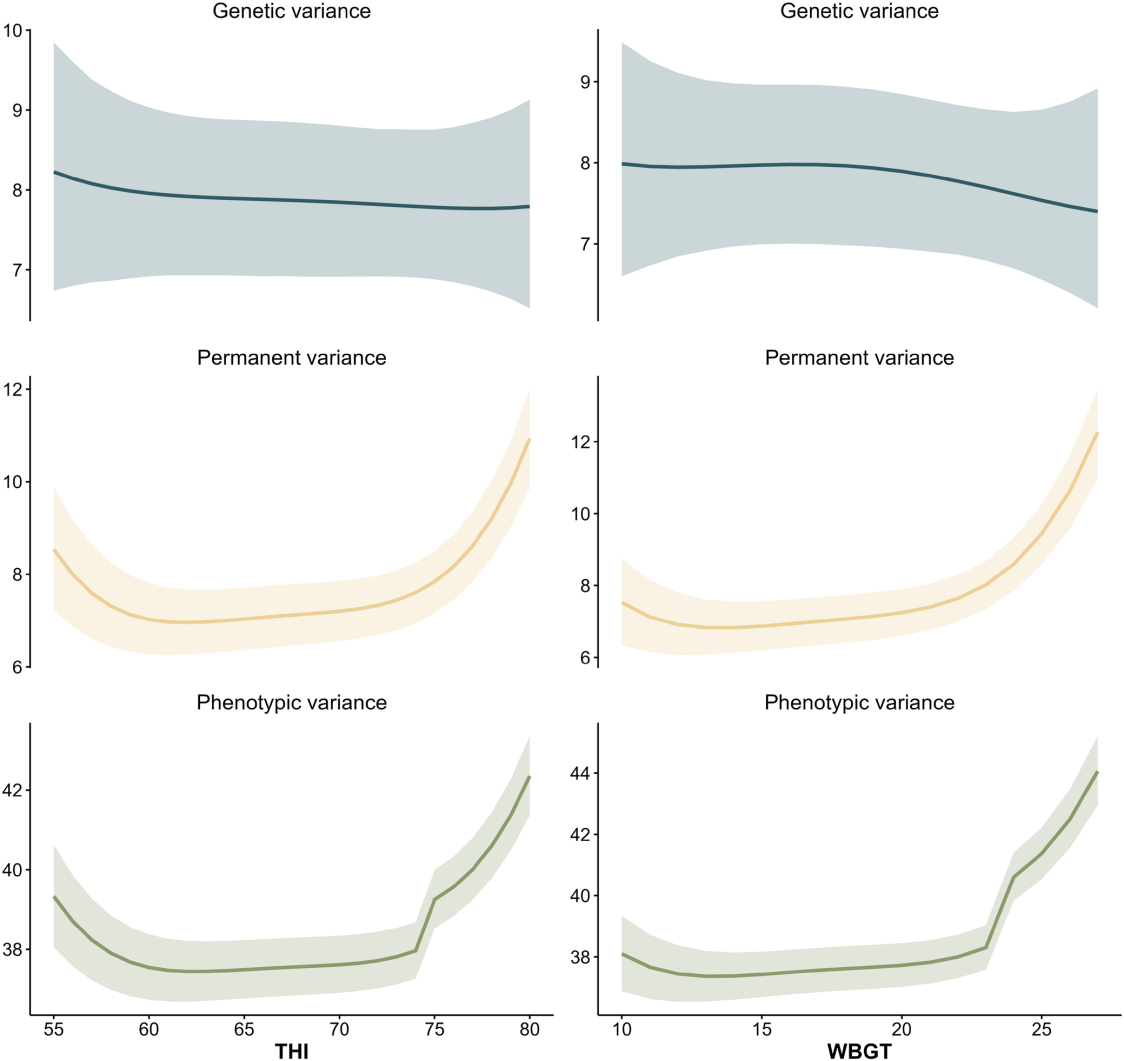
Posterior means (solid lines) and 95% highest posterior density intervals (shaded areas) for additive genetic, permanent environmental, and phenotypic variances of barrel racing time across temperature–humidity index (THI) and wet‐bulb globe temperature (WBGT) values, obtained from random regression models with quadratic Legendre polynomials. Estimates were expressed in seconds and multiplied by 10. [Colour figure can be viewed at wileyonlinelibrary.com]

**FIGURE 6:**
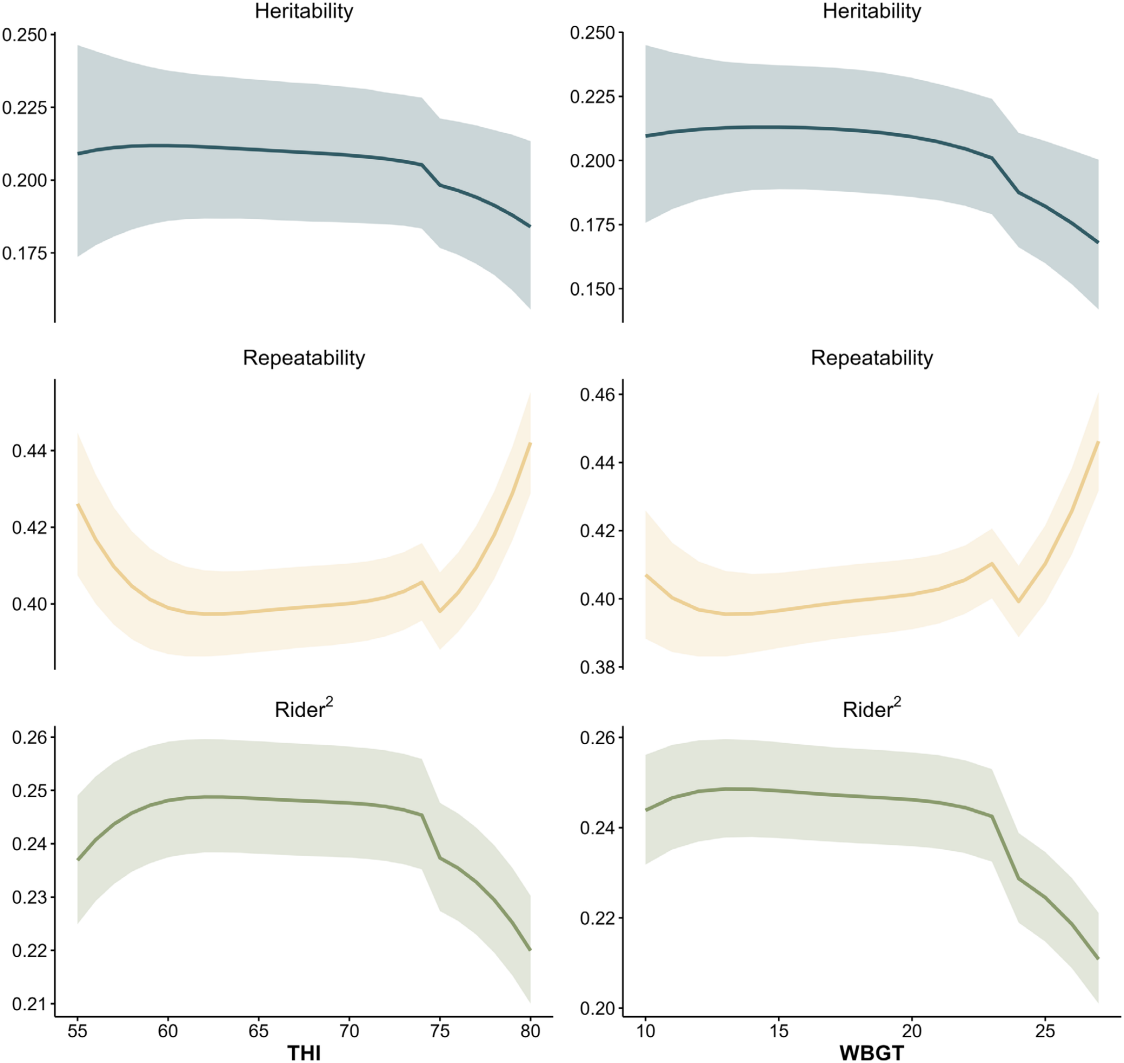
Posterior means (solid lines) and 95% highest posterior density intervals (shaded areas) for heritability, repeatability, and the rider effect (rider^2^, expressed as a proportion of phenotypic variance) of barrel racing time across temperature–humidity index (THI) and wet‐bulb globe temperature (WBGT) values, obtained from random regression models with quadratic Legendre polynomials. [Colour figure can be viewed at wileyonlinelibrary.com]

Figures 5 and 6 present the results derived from random regression models with quadratic Legendre polynomials fitted on THI (M6) and WBGT (M7). These models showed the best overall fit among the evaluated specifications, and the patterns of variation in the estimated variance components were consistent across both thermal indices. For this reason, only results from these models are displayed.

For THI, additive genetic variance changed little across the gradient, while permanent environmental variance declined from low to intermediate values and then increased toward higher THI. Phenotypic variance followed the same general pattern, with lower values in the mid‐range and higher values at elevated THI. For WBGT, additive genetic variance also varied within a narrow range, but permanent environmental and phenotypic variances increased more sharply at the upper end of the gradient. Overall, both indices displayed consistent trends, with WBGT showing a steeper increase in variability under higher thermal conditions, whereas THI exhibited a more gradual change across its range. Under the THI‐based model (M6), posterior means of the residual variance increased from 1.324 s^2^ (standard deviation = 0.003) under thermoneutral conditions to 1.430 s^2^ (0.007) under heat stress conditions. Similarly, under the WBGT‐based model (M7), residual variance rose from 1.329 s^2^ (0.003) in the thermoneutral zone to 1.510 s^2^ (0.011) under heat stress conditions.

Across both thermal indices, estimates of heritability, repeatability, and the rider effect as a proportion of the phenotypic variance (rider^2^) showed limited variation along the environmental gradients (Figure 6). For THI (M6), heritability was highly stable, averaging 0.21 across the gradient, with values close to 0.21 under thermoneutral conditions and declining modestly to 0.18 under extreme heat stress conditions. Repeatability remained nearly constant, with an overall mean of 0.41, increasing slightly from 0.40 in thermoneutral conditions to 0.44 at the highest THI levels. The rider contribution to phenotypic variance averaged 0.24, remaining close to 0.25 under thermoneutral conditions and decreasing marginally to 0.22–0.23 under extreme thermal conditions.

For WBGT (M7), mean heritability across the gradient was 0.20, with values around 0.21 in thermoneutral conditions and decreasing more clearly to 0.17 at the highest WBGT levels. Repeatability showed a small upward shift, averaging 0.41 overall, increasing from 0.40 in thermoneutral conditions to 0.45 under extreme heat stress. The rider effect averaged 0.24, declining from 0.25 in thermoneutral conditions to 0.21–0.22 at the upper end of the WBGT gradient.

**FIGURE 7:**
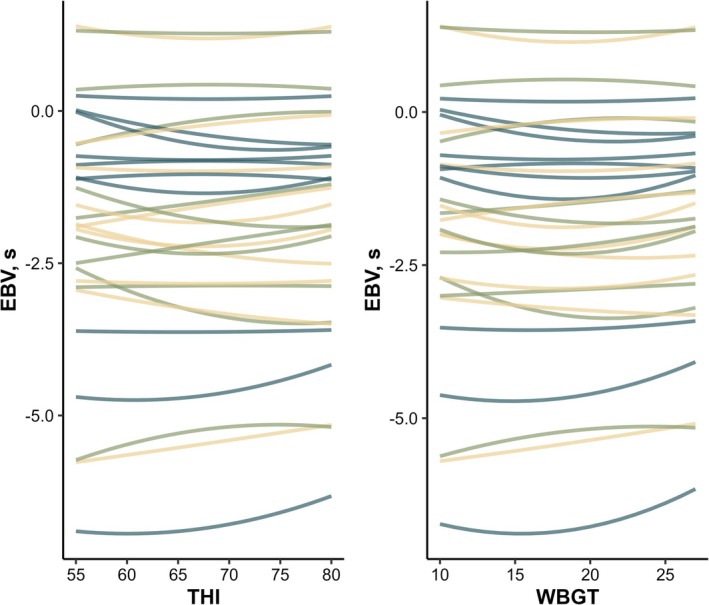
Posterior means of estimated breeding values (EBV) for barrel racing time of Quarter Horses across temperature–humidity index (THI, left panel) and wet‐bulb globe temperature (WBGT, right panel) values. Reaction norms are shown for the 5% most thermotolerant and 5% least thermotolerant stallions (with at least 10 progeny records). Estimates were obtained from random regression models with quadratic Legendre polynomials, expressed in seconds, and multiplied by 10. [Colour figure can be viewed at wileyonlinelibrary.com]

For both THI and WBGT, additive genetic correlations between the lowest values (55 for THI and 10 for WBGT) and higher levels remained extremely high across the entire gradients, declining only slightly from values close to unity at adjacent conditions to approximately 0.97 at the upper extremes. Overall, this pattern indicates strong genetic continuity of performance across thermal environments and suggests that genotype‐by‐environment interaction for BRT was limited in magnitude. Nevertheless, Figure 7, which displays the reaction norms of the 5% most thermotolerant and 5% least thermotolerant stallions identified in the auxiliary analysis for both THI and WBGT, reveals some individual variation in environmental sensitivity. In contrast, permanent environmental correlations showed a more pronounced decline as the thermal distance between conditions increased, decreasing steadily from values near 1.00 at neighbouring THI or WBGT levels to approximately 0.71–0.77 at the highest values.

### 3.6 Implications for Selection

Selection analyses were conducted using stallions with at least 10 progeny records for BRT. Under this criterion, 195 stallions were eligible for evaluation. From this group, the top 5% and top 10% of stallions were selected. When comparing thermoneutral and heat stress conditions within the same modelling framework, both the THI‐based model (M6) and the WBGT‐based model (M7) resulted in very similar sets of selected stallions. For the top 5%, 94.74% of the stallions were selected in common between thermoneutral and heat stress conditions. For the top 10%, the overlap increased to 97.37%, indicating a high level of selection consistency across thermal zones. Comparisons between models M6 and M7 within the same thermal zone also showed a high degree of agreement. Under thermoneutral conditions, selection based on the top 5% resulted in 100% overlap between the two models, while for the top 10%, 97.37% of the stallions were selected in common. Under heat stress conditions, agreement between M6 and M7 was slightly lower for the top 5%, with 89.47% of stallions selected in common, whereas for the top 10%, the overlap again reached 97.37%.

The auxiliary analysis revealed differences in overall genetic merit for BRT between animals classified as more and less thermotolerant. Using the THI‐based model (M4), the 100 most thermotolerant animals showed a mean EBV of −0.283 s, whereas the 100 least thermotolerant animals averaged −0.330 s, resulting in a mean difference of 0.047 s between groups. Using the WBGT‐based model (M5), the corresponding mean EBV was −0.277 s for the most thermotolerant and −0.407 s for the least thermotolerant animals, yielding a mean difference of 0.130 s between groups. Because lower EBV are favorable for BRT, the least thermotolerant animals showed more favorable overall genetic merit under the reference evaluation in both cases. These results suggest a possible antagonism between overall genetic merit for BRT and genetic tolerance to heat stress.

## 4 Discussion

Genetic tolerance to heat stress has been evaluated in several livestock species, with most studies identifying relevant genetic variation in performance responses that can be exploited in breeding programs (Carabaño et al. 2019). However, despite these advances, studies remain limited in horse breeds, revealing a clear gap in knowledge regarding genetic responses to heat stress (Dolores Gómez et al. 2015).

Studies evaluating thermal environments in animal performance datasets commonly report a strong dependence of derived thermal indices on the underlying temperature and humidity conditions (Rashamol et al. 2019; Yan et al. 2020). The very high Pearson correlation observed between THI and WBGT confirms that, in this dataset, both indices captured largely overlapping information on ambient thermal load. This close association is consistent with the fact that both indices are derived from similar climatic inputs (Michael et al. 2023).

Within this thermal context, segmented regression analyses identified clear breakpoints in the relationship between environmental conditions and barrel racing performance. For both indices, performance changed only marginally below the estimated thresholds, whereas steeper responses were observed once thermal conditions exceeded these limits. The similarity in the estimated breakpoints and in the post‐threshold response patterns for THI and WBGT indicates that the onset of performance sensitivity to heat stress was consistently captured by both indices, despite their different formulations. These findings support the existence of a definable critical thermal zone beyond which performance responses deteriorate more markedly. Although thermoregulatory mechanisms differ substantially between horses and cattle, several studies conducted in bovine populations (Bernabucci et al. 2014; Hammami et al. 2013; Santana et al. 2015) have reported THI‐based heat stress thresholds ranging from approximately 62–80 units, which overlap with the threshold values identified in the present study. More recently, Santana et al. (2026) evaluated Quarter Horses competing in sprint races and did not identify a heat stress threshold; instead, they reported a slight improvement in performance under higher thermal load. However, unlike the population analyzed here, the animals evaluated in that study competed exclusively at a single racetrack located in a region characterized by a relatively mild climate.

The geographic distribution of events complements the findings discussed above by demonstrating that competitions were widely distributed across Brazil, encompassing a broad range of climatic conditions. A substantial proportion of events and performance records occurred under thermal conditions exceeding the identified heat stress thresholds, indicating that exposure to heat stress was not restricted to isolated regions but was common across many municipalities, including areas in southern Brazil where the climate is generally regarded as milder. This broad spatial and environmental coverage reinforces the relevance of the identified thermal gradients and thresholds, as they represent conditions frequently encountered during competition rather than rare or exceptional scenarios. Specifically for horses, the thermoneutral zone has been reported to range from 5°C to 25°C (Morgan 1998), which is consistent with the occurrence of heat stress conditions during a substantial proportion of the barrel racing events analyzed in the present study.

These findings underscore the importance of greater awareness among organizers and participants of barrel racing events regarding potential risks to the health and welfare of both horses and riders. Monitoring thermal conditions during equestrian events is an established concern of international entities, such as the Fédération Equestre Internationale (https://www.fei.org/). However, similar practices do not appear to be consistently implemented in barrel racing events in Brazil. Nonetheless, more precise assessments of local thermal conditions should rely on appropriate on‐site measurements collected at the time and location of competition. In this context, it is also important to note that although the climatic data used in the present study were obtained from the NASA POWER platform and have been shown to be representative of surface‐based meteorological measurements, some degree of discrepancy may exist between locally measured conditions and values derived from satellite‐based or modelled data sources (Carrara et al. 2023; Monteiro et al. 2018).

The comparison of alternative model specifications indicates that accounting for continuous environmental variation provides a clear advantage when modelling BRT performance across thermal gradients. The superior performance of quadratic polynomial models for both THI and WBGT suggests that the relationship between thermal conditions and performance is inherently nonlinear. Thus, this relationship may be more appropriately captured by smooth reaction norms than by abrupt structural changes or constant effects. This finding is consistent with previous model comparison studies in dairy cattle, which showed that continuous polynomial functions provide a better fit to heat stress responses than threshold‐based models, as performance typically changes gradually across thermal gradients rather than following a strict broken‐line pattern (Carabaño et al. 2014). The poorer fit observed for segmented models indicates that, although thermal thresholds can be statistically identified at the population level, imposing discrete breakpoints in the genetic evaluation framework may oversimplify the underlying response across the full range of environmental conditions. Likewise, the weak performance of repeatability models highlights the limitations of assuming homogeneous environmental effects when substantial heterogeneity is present (Santana et al. 2025). Therefore, these results emphasize the importance of flexible modelling approaches that allow gradual changes in performance across thermal gradients, reinforcing the suitability of random regression models with polynomial functions for genetic analyses under varying thermal environments (Dolores Gómez et al. 2015).

Across both thermal indices, additive genetic variance remained relatively stable along the environmental gradients. This indicates that the genetic signal underlying barrel racing performance was largely preserved across a wide range of thermal conditions. In contrast, permanent environmental and phenotypic variances increased under higher thermal load, particularly at the upper end of the gradients. This pattern suggests that heat stress primarily magnified environmental sources of variability rather than inducing major changes in the magnitude of additive genetic effects. These variance dynamics have direct implications for genetic evaluation and selection. The modest declines in heritability from thermoneutral to extreme heat stress conditions observed in the present study primarily reflect the expansion of environmental variance rather than a reduction in additive genetic variance. Therefore, selection based on genetic merit remains feasible under challenging thermal environments. However, EBV accuracy across the thermal gradient was not directly evaluated in the present study. Because increased residual and permanent environmental variances under heat stress conditions may increase the uncertainty associated with EBV, future studies should assess whether the accuracy of genetic predictions changes under more extreme thermal conditions. Comparable patterns have been reported in equine populations evaluated under different competitive and environmental contexts. Dolores Gómez et al. (2015), working with Spanish trotter horses, reported heritability estimates for racing performance generally ranging from approximately 0.18–0.20 across thermal gradients, with only moderate reductions under warmer conditions. Consistent with these findings, Santana et al. (2026) evaluated sprint racing time in Brazilian Quarter Horses across temperature values using random regression models and reported heritability estimates varying slightly from 0.18 under milder thermal conditions to 0.15 in hotter environments, with additive genetic variance remaining detectable across the entire thermal gradient.

The slight reduction in additive genetic variance observed in the present study, as well as in the literature, may also be explained by a genetic antagonism or trade‐off between overall performance level and the specific response to heat stress. This interpretation was supported by the auxiliary analysis, in which the least thermotolerant animals had more favourable EBV for overall BRT under the reference evaluation. Both Dolores Gómez et al. (2015) and Santana et al. (2026) reported negative genetic correlations between the intercept and the slope of linear reaction norms. These results indicate that animals with superior genetic merit for general performance tended to show poorer responses under high thermal load. This pattern is a common finding across several livestock species, particularly in dairy cattle. The underlying explanation has been attributed to the fact that highly productive animals, or in the case of horses those with superior racing performance, generate greater metabolic heat and are therefore more susceptible to heat stress (Bernabucci et al. 2010). Although linear random regression models showed inferior fit to the BRT data and were not prioritized in the present analyses, the estimated genetic correlations between the intercept and the linear slope were consistently negative, ranging from −0.004 to −0.176 across models M2, M3, M4 and M5. In this context, selection focused exclusively on improved performance has been associated with a gradual decline in genetic tolerance to heat stress in livestock species (Carabaño et al. 2019, 2021; Santana et al. 2015) and, more recently, has also been documented in horses (Santana et al. 2026).

The high additive genetic correlations observed across THI and WBGT values indicate a strong genetic continuity of barrel racing performance across thermal environments. This suggests that largely the same set of genes influences performance under both thermoneutral and heat stress conditions. This pattern is consistent with previous findings in equine populations. In Quarter Horses competing in sprint races, Santana et al. (2026) reported posterior mean genetic correlations between race time at 12°C and all other temperature values ranging from 1.00 to 0.94, indicating limited genotype reranking across temperature conditions. Similarly, Dolores Gómez et al. (2015) reported genetic correlations greater than 0.90 for time per kilometre in Trotter Horses across thermal gradients. In the present study, the consistently high genetic correlations were accompanied by a high degree of agreement in stallion selection, with no substantial reranking observed among the top 5% or 10% sires. However, the reaction norms of the most and least thermotolerant stallions indicated detectable individual variation in environmental sensitivity. Thus, genetic variability in tolerance to heat stress was present, even though its impact on the ranking of top‐selected animals was limited. These findings indicate that the inclusion of genetic tolerance to heat stress in future breeding strategies may help sustain or enhance performance under increasingly challenging thermal environments, particularly in the context of ongoing climate change.

Although quadratic random regression models fitted on thermal indices provided the best statistical fit, the high genetic correlations across thermal environments and the generally similar reaction norms at the population level indicate that their practical advantage for routine animal ranking based specifically on thermal sensitivity may be limited in this population. When the breeding objective is restricted to overall BRT performance, more parsimonious alternatives may be appropriate. These may include the repeatability model with heterogeneous residual variance evaluated here or, preferably, an age‐based random regression model, as demonstrated by Santana et al. (2025) for the present population. Nevertheless, random regression models fitted on thermal indices provided additional inferential value by allowing the assessment of changes in additive genetic variance, heritability, genetic correlations, and animal ranking across the thermal gradient. Thus, their main contribution in the present study was not a substantial change in selection decisions, but rather a formal evaluation of genetic sensitivity to thermal load and the limited magnitude of genotype‐by‐environment interaction for BRT.

Based on the present results, no clear advantage of THI over WBGT, or vice versa, was identified in terms of overall model fit or selection outcomes. Both indices were highly correlated and led to largely consistent estimates of thermal thresholds, variance components, and stallion ranking. However, WBGT tended to capture slightly steeper changes in variance components and performance responses at the upper end of the thermal gradient, suggesting greater sensitivity to more extreme thermal conditions. In contrast, THI exhibited smoother and more gradual patterns across its range, which may favor its use in genetic models assuming continuous environmental gradients.

Despite its recognized limitations in fully characterizing thermal comfort (Arias and Mader 2023; Da Silva et al. 2007; Ouellet et al. 2021), THI remains the most widely used thermal index in genetic evaluations across livestock species due to its simplicity and ease of computation, relying solely on temperature and relative humidity. In contrast, WBGT is inherently more complex, requiring information on wet‐bulb and black globe temperatures, which are not always available and often need to be approximated. From a practical standpoint, these considerations suggest that THI may represent a more feasible and scalable option for routine genetic evaluations, while WBGT may offer added value when detailed environmental data are available and the focus is on characterizing responses under more extreme thermal conditions.

## 5 Conclusions

Both THI and WBGT captured largely overlapping environmental thermal information and produced consistent estimates of thermal thresholds, genetic parameters, and selection outcomes for barrel racing performance. Importantly, a considerable proportion of competitions occurred under heat stress conditions, underscoring the practical relevance of modelling performance across thermal gradients. Models accounting for continuous thermal variation provided a superior fit, indicating that performance responses to heat stress are best described by smooth reaction norms rather than discrete thresholds. Although clear performance breakpoints were identified for both indices, additive genetic variance and genetic correlations remained high across thermal gradients, with no evidence of substantial animal reranking. This indicates limited genotype‐by‐environment interaction for BRT. From a practical perspective, THI and WBGT performed similarly in genetic evaluations. WBGT was slightly more sensitive under extreme thermal conditions, whereas THI offered greater simplicity and broader data availability. Overall, these results support the use of random regression models to sustain genetic progress in barrel racing performance under increasingly challenging thermal environments.

## Funding

The authors have nothing to report.

## Conflicts of Interest

The authors declare no conflicts of interest.

## Data Availability

The data that support the findings of this study are available from the corresponding author upon reasonable request.
